# Educational efficacy of medical humanities in empathy of medical students and healthcare professionals: a systematic review and meta-analysis

**DOI:** 10.1186/s12909-023-04932-8

**Published:** 2023-12-06

**Authors:** Xin Zhang, Hui-fang Pang, Zhiguang Duan

**Affiliations:** 1 School of Mangement, Shanxi Medical University, TaiYuan, 030001 China; 2 Shanxi Cardiovascular Disease Hospital, TaiYuan, 030024 China

**Keywords:** Medical humanities, Empathy, Humanities education, Procedure, Time factors

## Abstract

**Background:**

Medical humanities education is an important part of medical education. The purpose of this study was to determine the effectiveness of medical humanities in improving empathy among medical students and healthcare professionals.

**Methods:**

PubMed, Embase, EBSCO-ERIC, Web of Science were searched systematically for studies in the English language. The last retrieval date is May 1, 2023. Best Evidence Medical Education (BEME) Global Rating Scale and Kirkpatrick-based results were used to evaluate the quality of literature. In this study, a meta-analysis of continuous data was conducted.

**Results:**

The pooled results by single-arm test meta-analysis showed a benefit with medical humanities programs in empathy (SMD 1.33; 95% CI 0.69–1.96). For single-arm trials of medical humanities program interventions of less than 4 months, 4 months to 12 months, and more than one year, the standardized mean differences(SMD) between post-test and pre-test were 1.74 (*P* < 0.05), 1.26 (*P* < 0.05), and 0.13 (*P* = 0.46), respectively. The results showed a significant difference in the effect of medical humanities programs on male and female empathy (SMD − 1.10; 95% CI -2.08 – -0.13). The SMDs for the study of course, the course combined reflective writing, and the course combined reflective writing and practice as intervention modalities for medical humanities programs were 1.15 (*P* < 0.05), 1.64 (*P* < 0.05), and 1.50 (*P* < 0.05), respectively.

**Conclusion:**

Medical humanities programs as a whole can improve the empathy of medical students and health professionals. However, different intervention durations and different intervention methods produce different intervention effects.

**Supplementary Information:**

The online version contains supplementary material available at 10.1186/s12909-023-04932-8.

## Background

The concept of empathy is widely used in the health care field. That’s because empathy is often associated with good healthcare outcomes [1], including good chronic disease management [2], reduced severity of illness [3], and reduced symptoms of post-traumatic stress disorder [4].

But as our understanding of big data and artificial intelligence expands in the medical field, the experience of patients is marginalized [5]. And medical informatization further compounds the information asymmetry between physicians and patients [6]. These factors contribute to patients perceiving a deficit of empathy in the clinician-patient relationship. Empathy is generally understood as a cognitive and affective ability to understand the thoughts and feelings of others [7]. This definition assumes that empathy is acquired through interacting appropriately with others [8]. In recent years, medical humanities have been proposed as a solution to the “negative” problems of medicine [9]. So does medical humanities play a role in promoting empathy among doctors?

Medical humanities have been proposed as an activity that might improve empathy in medical students, by fostering skills such as the interpretation of narratives, and the ability to manage situations where there is no single correct answer [10]. There is a lot of research on the role of medical humanities in empathy. The results of Huang et al. showed that some students believed that the most important role of medical humanities training was to improve empathy [11]. Ronan et al. used a medical humanities education program in the form of cartoons to intervene with residents and showed an effective increase in resident empathy [12]. Satendra et al. intervened in medical students’ empathy with medical humanities programs such as Theatre of the Oppressed, Reflective Writing, and others, and showed that they could effectively improve medical students’ empathy [13]. But other studies on the role of medical humanities on empathy have come to a different conclusion. A study by Graabækd et al. showed no effect of medical humanities programs in the form of reading on medical staff empathy [14]. Cédric’s findings also showed no significant difference in empathy between the narrative medicine group and the control group [15]. In this paper, we used meta-analysis to examine the impact of a medical humanities education program on the empathy of medical students and healthcare professionals. We also compared the effects of the intervention across different intervention times, across different program types, and gender. It will provide evidence support for medical humanities to improve empathy.

## Methods

Our current work is consistent with the Preferred Reporting Items for Systematic Reviews and Meta-Analyses guidelines. Collected and included in this study were published studies.

### Search strategy

On April 27, 2023, we searched three databases (Web of Science, PubMed EBSCO-ERIC, and Embase). The search strategy for this study can be found in Appendix 1.

### Study inclusion criteria

The study inclusion criteria included that the (i) intervention was a medical humanities program, (ii) studies intervention was on medical students and medical workers’ empathy, (iii) studies had a quantitative assessment of empathy, (iv) and studies published in the English language.

### Study selection

Studies was screened in two steps: (i) a title and abstract review phase, (ii) and a full-text review phase. If a paper meets the inclusion criteria in phase (i), the paper was reviewed in phase (ii). Two reviewers (ZX and PHF) reviewed the paper at phase (i) and phase (ii), respectively. Any disagreements were resolved either through discussion or consultation with a third reviewer (DZG).

### Data extraction

Two reviewers (ZX and PHF) were assigned to extract the study characteristics independently. Demographic data extracted included: author name, title, date of publication, location of study, journal, study type, sample size, and intervention time. Data extraction items for study aims included: measurement tools, study procedures, empathy scores, and Gender-specific empathy scores. Any disagreements were resolved either through discussion or consultation with a third reviewer (DZG).

### Study quality assessment

Study quality evaluation was performed by ZX and DZG, who classified the articles according to the Best Evidence Medical Education (BEME) Global Rating Scale and Kirkpatrick-based results (Appendix 2). Any disagreements between the two reviewers were resolved through discussion.

### Data analysis

In studies with single-arm studies, the outcome of post-intervention was used as the experimental group, and the outcome of pre-intervention as the control group for quantitative synthesis. In the Rcts studies, the medical humanities programs were regarded as an experimental group whereas the other served as the control for quantitative synthesis. In the randomized pre and post intervention design controlled studies, the outcome (medical humanities programs) of post-intervention was used as the experimental group, and the outcome (medical humanities programs) of pre-intervention as the control group for quantitative synthesis, the outcome (medical humanities programs) of post-intervention was used as the experimental group, and the outcome (control group) of post-intervention as the control group for quantitative synthesis.

We calculated a pooled standardized mean difference (SMD) and 95% confidence intervals (CI). Whenever heterogeneity was not detected (*P* for heterogeneity > 0.05), a fixed effect model was used; otherwise, a random effect model was used. If the p-value for heterogeneity was < 0.1 or I^2^ was > 50%, the studies were considered heterogeneous. Analysis of the data was performed using Review Manager software version 5.4.

## Results

**Fig. 1:**
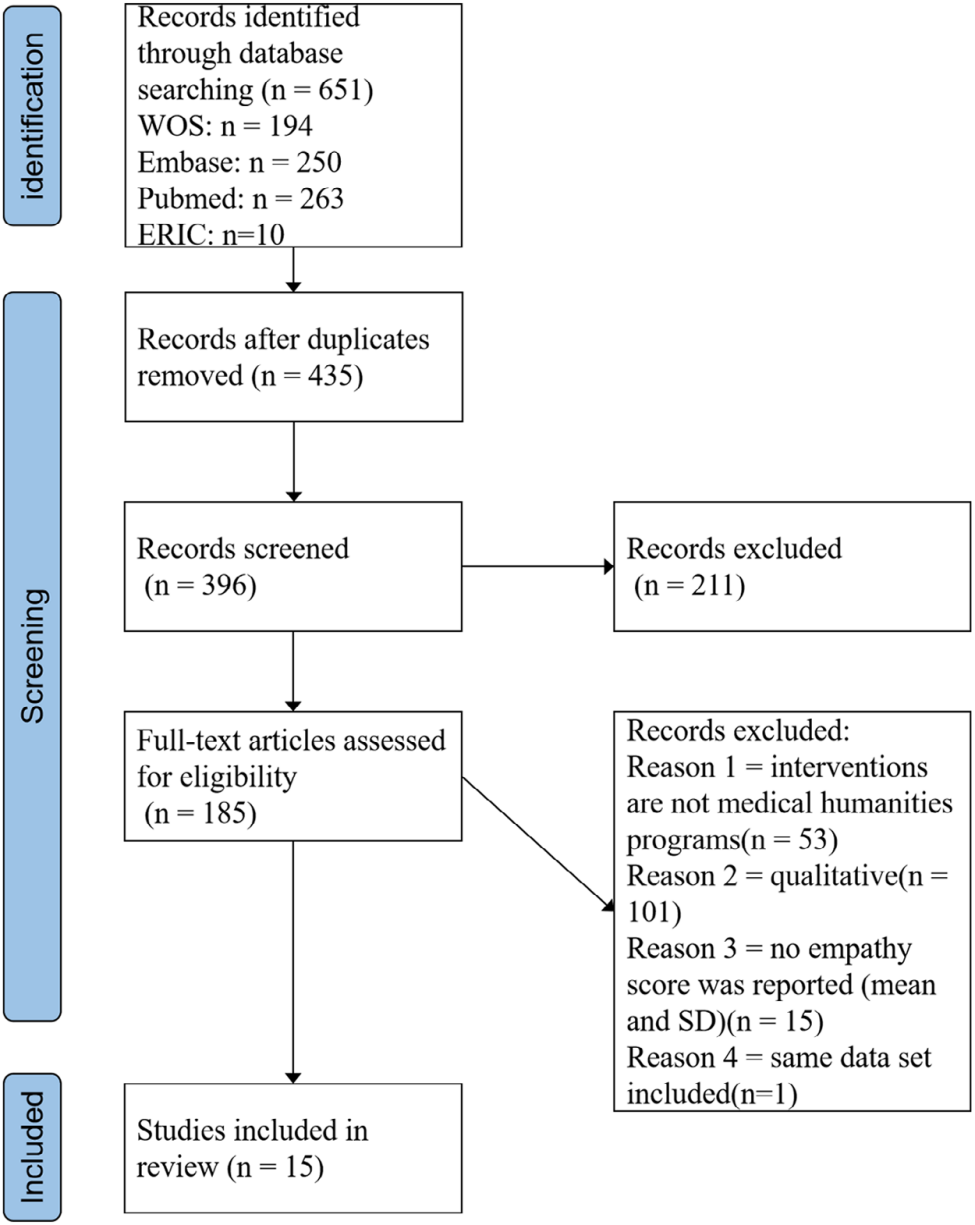
Flow chart of the study selection process

The selection process is displayed In Fig. 1. The final meta-analysis included fifteen studies. A comprehensive description of each study is provided in Appendix 3.

### Characteristic of included trials

Twelve of the 15 included studies were conducted with medical students and 3 with healthcare professionals. The majority of studies assessed the impact of medical humanities programs on the empathy of Chinese medical students and healthcare professionals (47%), followed by U.S. medical students or healthcare professionals (27%). The Jefferson Scale of Empathy (JSE) was used to measure empathy scores in most of the studies (80%). Medical humanities program interventions lasted less than 4 months in 10 studies, 4 months-1 year in 3 studies, and > 1 year in 2 studies. Of the 15 studies, 13 studies used before-and-after controlled trials, and 5 studies combined before-and-after controlled and RCT trials. A comprehensive description of each study is provided in Table 1.

**Table 1:** Characteristics of included studies

Study	Design	Intervention measures/intervention time	Population/Sample	Measurement Tool	Empathy score, mean(SD)
Bahadur et al.(2015), Nepal [31]	Single arm pre post study	Course AND Expose to care practice AND Reflective writing practice/8 weeks	Medical students/n = 65	JSE-S	**Pre-**: 105.52(10.45) **Post-**: 116.29(9.02)
Johanna et al.(2004), USA [19]	Single arm pre post study	Course/3 Months	Medical students/n = 16	ECRS	**Pre-**: 92.3(8.2) **Post-**: 94.6(8.9)
Xue et al. (2023), China [20]	Single arm pre post study, RCT	Course AND Reflective writing practice/12 Months	Nursing students/n = 85	JSPE-NS	**All-Post**: I-G:99.4(15.7),C-G:92.2(14.6) **I-G**:Pre-: 89.6(14.0),Post-:99.4(15.7) **C-G**: Pre-: 88.7(11.9),Post-: 92.2(14.6)
Michal et al. (2020), Israel [25]	Single arm pre post study	Course/3 years	Medical students/n = 262	JSPE-S	**Pre-**: 114.40(11.32) **Post-**: 112.75(14.19)
Chen et al. (2017), Taiwan, China [32]	Single arm pre post study	Course AND Reflective writing practice/2 Months	Healthcare professionals/n = 142	JSE-HP	**Pre-(110)**: 111.1(1.4) **Post-(100)**: 116.2(1.6) **1.5years(90)**: 116.0(1.6)
Yang et al. (2013), Taiwan, China [33]	Single arm pre post study	Exposure to visual art/4 Months	Medical students/n = 113	JSPE	**Pre-(110)**: 110.92(10.27) **Post-(110)**: 111.30(11.57)
Cédric et al.(2020), France [15]	RCT	Course AND Reflective writing practice/2 Months	Medical students/n = 362	JSPE-MS	**C-G**: 110.1(11.9) **Balint groups**: 111.0(9.1) **Narrative medicine**: 110.7(9.3)
Chen et al. (2022), Taiwan, China [21]	RCT	Narrative/Storytelling/9 Months	Medical students/n = 207	JSPE	**C-G**: 69.4(11.3) **I-G**: 69.7(11.9)
Brian et al. (2020),USA [34]	Single arm pre post study	Course AND Expose to care practice AND Reflective writing practice/4 Months	Medical students/n = 34	JSE	**Pre-**: 5.75(0.1) **Post-**: 6.05(0.09)
Haley et al. (2018), USA [23]	Single arm pre post study	Course AND Reflective writing practice/1 years	Medical students/n = 25	Self-made questionnaire(contains 9 items)	**Pre-(25)**:3.25(0.42) **Post-(22)**:3.82(062)
Lon J et al. (2021), USA [35]	Single arm pre post study	Course/4 Months	Medical students/n = 60	JSE	**Pre-**: 109.10(1.28) **Post-**:112.22(1.07)
Yang et al. (2018), China [36]	Single arm pre post study, RCT	Course AND Expose to care practice AND Reflective writing practice/30Months	Nursing students/n = 163	JSE	**T1(pre-)**: G1:104.08(12.43) G2:104.59(13.48) G3:104.42(14.11) **T2(theoretical education ends)**: G1:104.06(11.75) G2:107.45(13.34) G3:107.07(14.08) **T3(clinical practice education ends)**: G1:104.79(11.82) G2:107.91(13.01) G3:110(13.30)
Zhao et al. (2023), China [24]	Single arm pre post study	Course/2 Months	Healthcare professionals/n = 116	JSPE-S	**Pre-**: 110.6(12.1) **Post**: 122.6(9.0)
Saeideh et al. (2020), Iran [26]	Single arm pre post study, RCT	Course AND Reflective writing practice/3 Months	Medical students/n = 135	JSPE	**C-G**: Pre-:75.86(8.50),Post:76.35(7.99) **I-G**:Pre-:73.90(8.59),Post:94.90(4.47)
Lu et al. (2023), China [22]	Single arm pre post study	Course/6 Months	Nursing/n = 101	The Interpersonal Response Index Scale	**Pre-**:21.64(2.3) **Post**:26.71(3.1)

I-G: Intervention group, C-G: Control group

Study quality was evaluated using BEME and Kirkpatrick-based results, with 9 studies having a BEME of 3, 6 studies having a BEME of 4, 11 studies having a Kirkpatrick-based result of 2a, and 4 studies having a Kirkpatrick-based result of 2b. A comprehensive BEME and Kirkpatrick-based results for each study are provided in Appendix 4.

### The impact of medical humanities programs on empathy

**Fig. 2:**
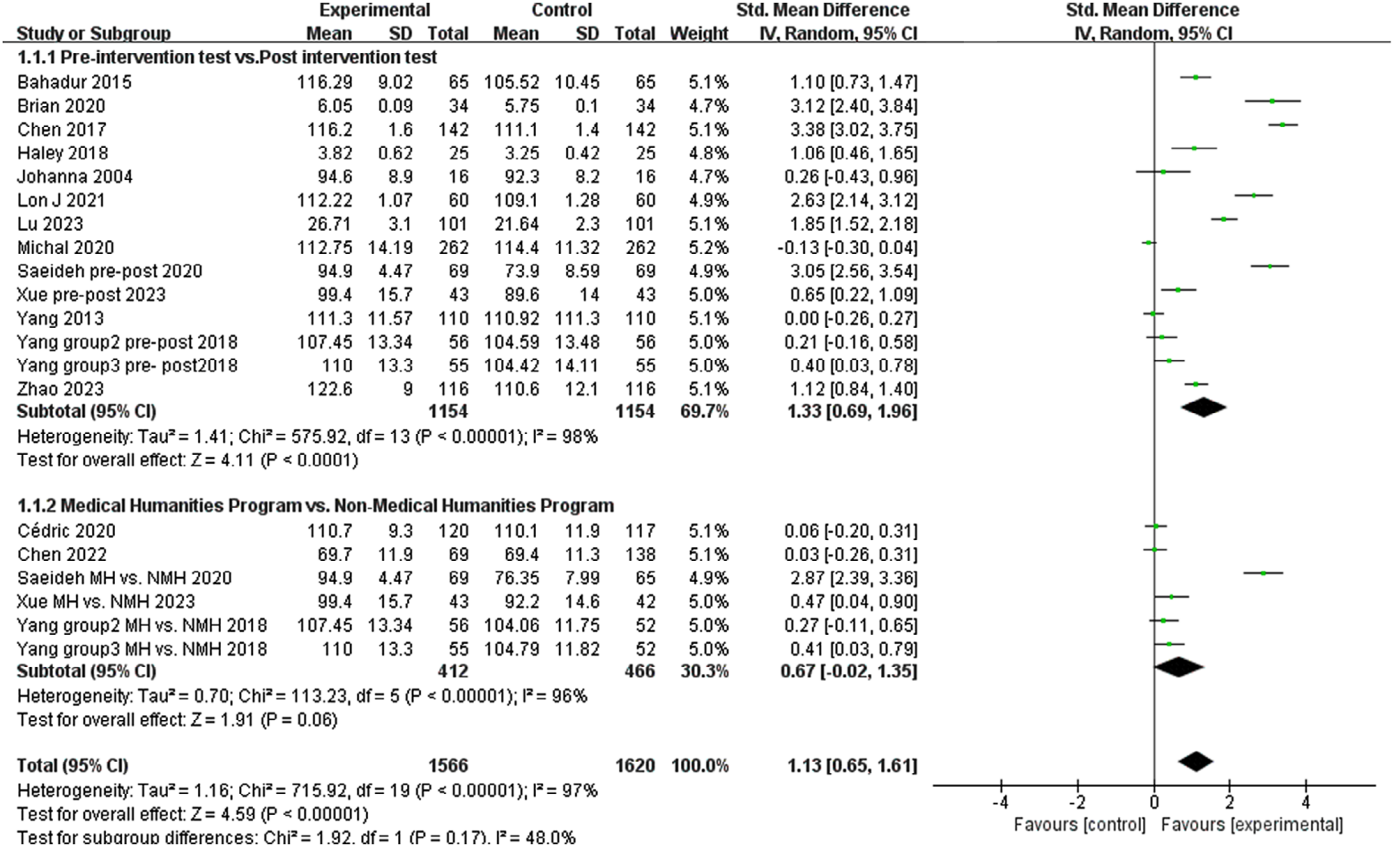
Forest plot showing the empathy of medical student or healthcare professionals. MH: Medical humanities program, NMH: Non-Medical humanities program

Fourteen single-arm pre-post studies assessed the impact of medical humanities programs on empathy. Nine of the 14 per-post studies showed an increase in medical student and healthcare professionals empathy scores after the medical humanities program intervention. The pooled results by meta-analysis showed a large benefit with medical humanities programs in empathy (SMD 1.33; 95% CI 0.69–1.96) and the evidence of heterogeneity with an I2 = 98% was also shown (Fig. 2).

Five RCTs studies investigated the impact of the presence or absence of a medical humanities program on the empathy of medical students and healthcare professionals. Meta-analysis results showed no difference in empathy (SMD 0.67; 95% CI -0.02–1.35) (Fig. 1) between medical students and healthcare professionals who participated in the medical humanities program and those who did not.

### Effect of medical humanities program intervention time on empathy

**Fig. 3:**
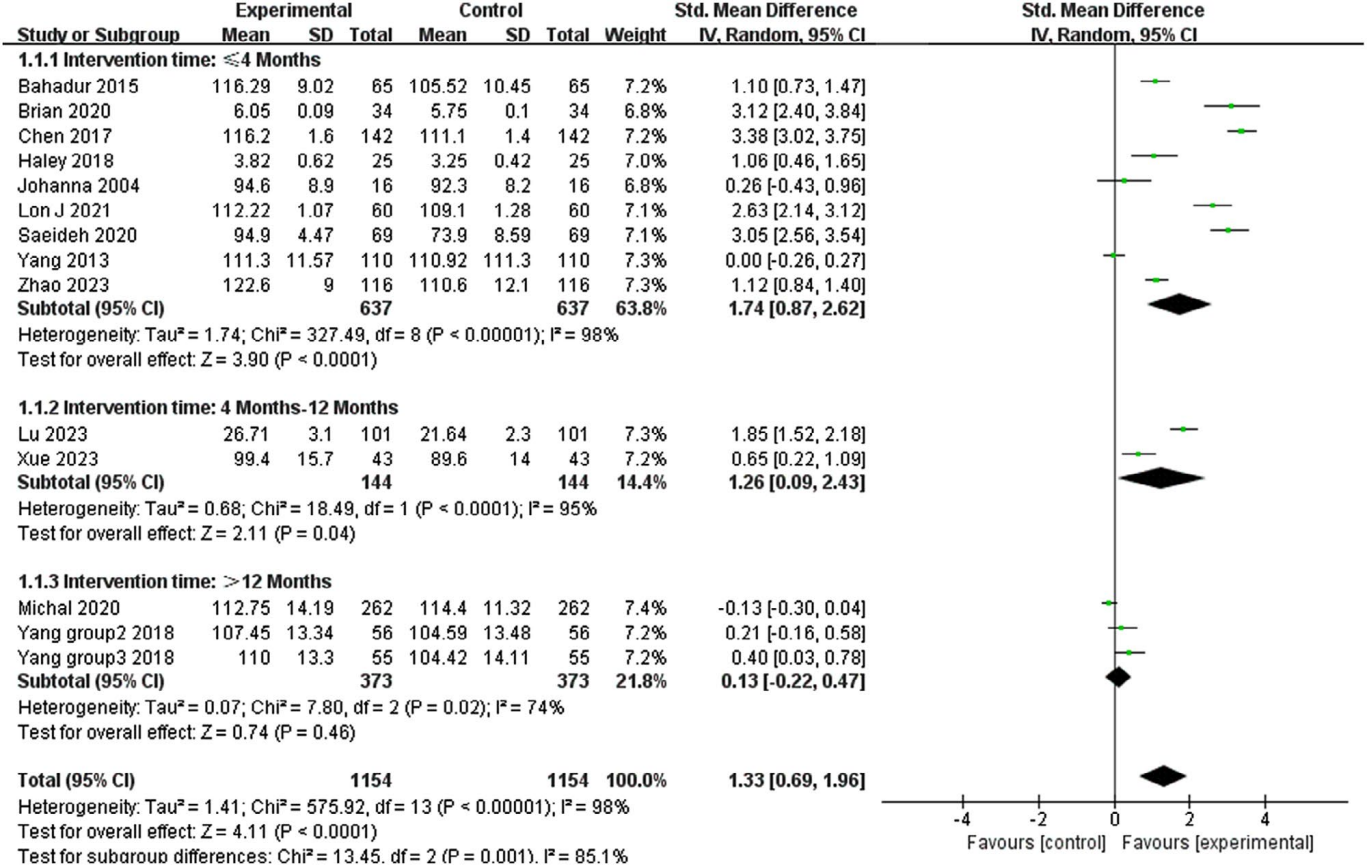
Effect of medical humanities program intervention time on empathy

**Fig. 4:**
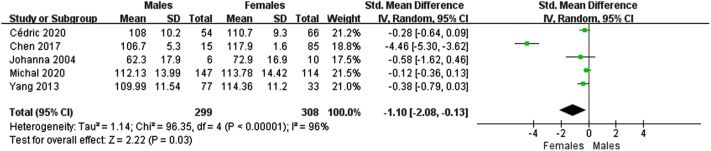
Forest plot showing the empathy of females and males

Nine studies evaluated the impact of medical humanities programs on empathy over a period of 4 months or less. The results of the meta-analysis showed that medical humanities programs of up to 4 months (SMD 1.74; 95% CI 0.87–2.62)(Fig. 2) and 4 to 12 months (SMD 0.73; 95% CI -0.58–2.04)(Fig. 2) have a tremendous benefit in enhancing the empathy of medical students or healthcare professionals. In contrast, medical humanities programs with intervention durations of more than 12 months had no effect on the improvement of empathy (SMD 0.13; 95% CI -0.22–0.47)(Fig. 3).

### Males vs. females

Five studies compared the effects of medical humanities programs on the empathy of medical students or healthcare professionals across gender. The results showed a significant difference in the effect of medical humanities programs on male and female empathy (SMD − 1.10; 95% CI -2.08 – -0.13)(Fig. 4). Thus medical humanities programs benefit females more than males in terms of empathy.

### Effects of different intervention types on empathy

**Fig. 5:**
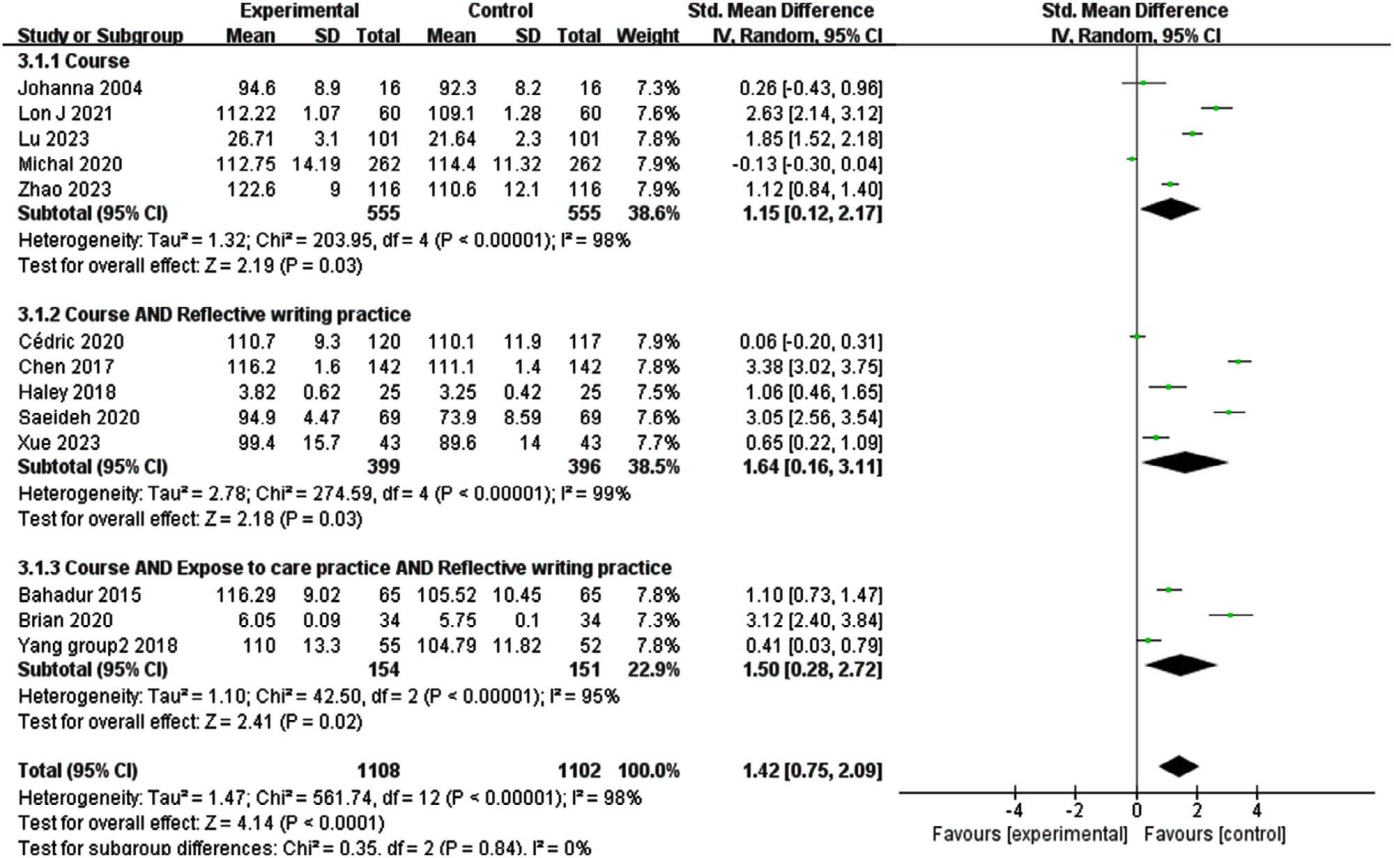
Forest plot showing the empathy of different intervention types

Course, reflective writing, and care practice are the most used interventions in medical humanities programs. Five studies of medical humanities programs adopted the course intervention, and meta-analysis results showed that the course had a significant effect on improving empathy (SMD 1.15; 95% CI 0.12–2.17) (Fig. 4). Three studied medical humanities programs adopted a course combined reflective writing intervention, and meta-analysis results showed that this intervention had a significant effect on improving empathy (SMD 1.64; 95% CI 0.16–3.11) (Fig. 4). Three studied medical humanities programs adopted a course that combined reflective writing and clinical practice intervention, and meta-analysis results showed that this intervention had a significant effect on improving empathy (SMD 1.50; 95% CI 0.28–2.72) (Fig. 4). And there was no significant difference in the effect of the above three interventions on empathy (Chi^2^ = 0.35, *P* = 0.84) (Fig. 5).

## Discussion

To our knowledge, this is the first published systematic review of the effects of medical humanities programs on empathy and the first to analyze the effects of different medical humanities programs and different intervention times on empathy separately. Several systematic reviews have summarised the impact of medical humanities programs on empathy. In a 2017 systematic review, it was noted that narrative medicine is beneficial for improving empathy in healthcare workers, but not enough to provide clinical evidence to support it due to a lack of large-scale studies. [16] In a 2019 systematic review, mention was made of the fact that narrative medicine can improve medical students’ empathy, thus helping them to build a harmonious doctor-patient relationship. However, due to the lack of relevant longitudinal studies, the long-term intervention effects of medical humanities programs are less certain. [17] A 2022 systematic review on the impact of medical humanities education on medical student learning outcomes in Taiwan, China, also mentioned that medical humanities education can improve medical students’ empathy. [18] Although these reviews mention that medical humanities can improve medical students’ and healthcare professionals’ empathy, none of these reviews provide a detailed analysis of the duration and content of interventions in medical humanities programs.

The findings of this study have important implications for medical humanities education, practice, and future research. The results of this review indicate that medical humanities programs show positive effects in enhancing empathy in both medical students and medical staff. There are two main reasons why medical humanities programs can increase empathy in medical students and medical staff. The former is that it can help medical students and medical staff to reflect on themselves, increase their professional identity, and improve their empathy. [19–22] The second is that it can help them to think from the patient’s point of view and better understand the patient. [20, 23, 24] The results of several RCTs showed no effect of medical humanities programs on improving empathy. This may be because the subjects of these studies were medical students in clinical placements. These students, new to the clinic lacking appropriate role models and with a strong need for technology, would not consider empathy an important component. [15, 25] Secondly, most medical schools emphasize humanistic education for medical students, so students in both the control and experimental groups may have had previous training in medical humanities. [26].

The findings of this review specifically suggest that the longer the intervention period of the medical humanities program the less effective the empathy enhancement. One possible explanation for this is that when students begin a course, they first experience a honeymoon period. Afterward, as the course becomes more standardized, students gradually become bored with the course and it becomes less effective. [27] Another explanation is that medical students have a high level of burnout that increases with the duration of study. [28] A third explanation is that as medical students enter the internship period, empathy also builds up fatigue, which causes it to decrease. [24].

The findings show that medical humanities programs have a higher effect on empathy in females than in males. It has been argued that women tend to be more receptive to signs of emotion than men. [29] Evidence also shows that female medical professionals can respond more quickly to medical humanities interventions compared to men. [30].

The results of this study also indicate that theoretical education combined with practical education is more effective than theoretical education alone in enhancing empathy. During practice, medical students and medical staff can have effective interaction and deepen the new knowledge and skills they have learned. [20] Moreover, in practice, they can have full contact with patients and have a deeper understanding of them. [24] Problems encountered in practice are unique and can form summative feedback for medical staff, which helps medical staff to reflect on themselves. [21].

### Strengths and limitations

To our knowledge, this is the first meta-analysis of the impact of medical humanities programs on empathy. The strengths of this study include the following: (1) it was a comprehensive literature search of relevant studies; (2) it compared the effects of different intervention times on empathy; and (3) it compared the effects of different types of medical humanities programs on empathy.

Limitations of this systematic review include: (1) significant heterogeneity in the meta-analysis, which may be due to differences in the content, approach, and process of various types of medical humanities programs; (2) fewer studies used arts-based interventions to improve empathy, failing to form comparisons with other types of interventions; (3) the largest number of included studies were in China and the United States, and uneven distribution of geography, ethnicity, etc. may also affect the generalizability of the results; and (4) most studies did not have quality control regarding medical humanities programs, and differences in the quality of the studies may also lead to biased findings.

### Implications and conclusion

This study has important implications for the application of medical humanities programs to practice. Medical humanities programs as a whole can improve the empathy of medical students and health professionals. However, different intervention durations and different intervention methods produce different intervention effects. This suggests that the duration and the manner of the intervention are important factors that influence medical humanities programs to improve empathy among medical students or medical staff. Moreover, the medical humanities program should have a different focus for medical students and medical staff of different genders.

### Electronic supplementary material

Below is the link to the electronic supplementary material.

**Supplementary Material 1**: Appendix 1 Search strategy

**Supplementary Material 2**: Appendix 2 Best Evidence Medical Education (BEME) coding scheme for strength of evidence and Kirkpatrick-based outcomes

**Supplementary Material 3**: Appendix 3 Characteristics of included studies

**Supplementary Material 4**: Appendix 4 BEME and Kirkpatrick-based results

## Data Availability

The datasets generated and analysed during the current study are available upon reasonable request with the corresponding author.

## References

[1] Sanders JJ, Dubey M, Hall JA, Catzen HZ, Blanch-Hartigan D, Schwartz R (2021). What is empathy? Oncology patient perspectives on empathic clinician behaviors. Cancer.

[2] Etingen B, Miskevics S, LaVela SL (2016). Assessing the associations of patient-reported perceptions of patient-centered Care as Supplemental measures of Health Care Quality in VA. J Gen Intern Med.

[3] Rakel D, Barrett B, Zhang Z, Hoeft T, Chewning B, Marchand L (2011). Perception of empathy in the therapeutic encounter: effects on the Common Cold. Patient Educ Couns.

[4] Moss J, Roberts MB, Shea L, Jones CW, Kilgannon H, Edmondson DE (2019). Healthcare provider compassion is associated with lower PTSD symptoms among patients with life-threatening medical emergencies: a prospective cohort study. Intensive Care Med.

[5] Chen W, Feng Y, Fang J, Wu J, Huang X, Wang X (2020). Effect of trust in primary care physicians on patient satisfaction: a cross-sectional study among patients with Hypertension in rural China. BMC Fam Pract.

[6] John G. Compassion, medical humanities and medical education — University of Edinburgh research explorer. Education for Primary Care. 2018.

[7] Cao EL, Blinderman CD, Cross I (2021). Reconsidering Empathy: An Interpersonal Approach and Participatory Arts in the Medical humanities. J Med Humanit.

[8] Gilbert PO. Compassion: Universally Misunderstood. Huffington Post. 2015.

[9] Shalev D, McCann R (2020). Can the medical humanities make trainees more compassionate? A neurobehavioral perspective. Acad Psych.

[10] Karnieli-Miller O, Vu TR, Frankel RM, Holtman MC, Clyman SG, Hui SL (2011). Which experiences in the hidden curriculum teach students about professionalism?. Acad Med.

[11] Huang C-D, Jenq C-C, Liao K-C, Lii S-C, Huang C-H, Wang T-Y (2021). How does narrative medicine impact medical trainees’ learning of professionalism? A qualitative study. BMC Med Educ.

[12] Lk R, Mk C. A novel graphic medicine curriculum for resident physicians: boosting empathy and communication through comics. J Med Humanit. 2020;41. 10.1007/s10912-020-09654-2 32809157

[13] Singh S, Barua P, Dhaliwal U, Singh N (2017). Harnessing the medical humanities for experiential learning. Indian J Med Ethics.

[14] Graabæk T, Rasmussen AJ, Mai AM, Rossing C, Hedegaard U, Can pharmacists improve their patient communication by reading fiction? Narrative medicine in pharmacy practice ? A feasibility study— University of Southern Denmark. Spain; Ulla.

[15] Lemogne C, Buffel du Vaure C, Hoertel N, Catu-Pinault A, Limosin F, Ghasarossian C (2020). Balint groups and narrative medicine compared to a control condition in promoting students’ empathy. BMC Med Educ.

[16] Barber S, Moreno-Leguizamon CJ (2017). Can narrative medicine education contribute to the delivery of compassionate care? A review of the literature. Med Humanit.

[17] Milota MM, van Thiel GJMW, van Delden JJM (2019). Narrative medicine as a medical education tool: a systematic review. Med Teach.

[18] Hoang B, Monrouxe L, Chen K, Chang S, Chiavaroli N, Mauludina Y et al. Medical Humanities Education and its influence on students’ outcomes in Taiwan: a systematic review. Front Med. 2022;9. 10.3389/fmed.2022.857488 PMC9150274 35652071

[19] Shapiro J, Morrison E, Boker J (2004). Teaching Empathy to First Year Medical students: evaluation of an Elective Literature and Medicine Course. Educ Health: Change Learn Pract.

[20] Xue M, Sun H, Xue J, Zhou J, Qu J, Ji S (2023). Narrative medicine as a teaching strategy for nursing students to developing professionalism, empathy and humanistic caring ability: a randomized controlled trial. BMC Med Educ.

[21] Chen C-H, Wang S-J, Yeh W-Y, Wu C-L, Wang YA, Chen C-F (2022). Evaluating Teaching Effectiveness of Medical Humanities in an Integrated Clerkship Program by a novel prospective propensity score matching Framework. Int J Environ Res Public Health.

[22] Lu N, Ma Z, Shi Y, Yao S, Zhang L, Shan J et al. A narrative medicine-based training program increases the humanistic care quality of new nurses in cancer hospital. Precision Med Sci. 2023;12.

[23] Ilcewicz HN, Poirier TI, Pailden J (2018). Use of mixed-methods approach to assess the impact of a pre-professional health humanities honors course on developing interpersonal skills. Curr Pharm Teach Learn.

[24] Zhao J, Xiantao O, Li Q, Liu H, Wang F, Li Q (2023). Role of narrative medicine-based education in cultivating empathy in residents. BMC Med Educ.

[25] Lwow M, Canetti L, Muszkat M. Gender differences in the effect of medical humanities program on medical students’ empathy: a prospective longitudinal study. BMC Med Educ. 2020;20. 10.1186/s12909-020-02333-9 PMC7653998 33167937

[26] Daryazadeh S, Adibi P, Yamani N, Mollabashi R (2020). Impact of a narrative medicine program on reflective capacity and empathy of medical students in Iran. J Educ Eval Health Prof.

[27] Stacey G, Wilson C, Reddy H, Palmer C, Henderson J, Little H (2018). Diagnosing and treating Enquiry Based Learning fatigue in Graduate Entry nursing students. Nurse Educ Pract.

[28] Zhang J-Y, Shu T, Xiang M, Feng Z-C (2021). Learning burnout: evaluating the role of Social Support in Medical Students. Front Psychol.

[29] Kret ME, De Gelder B (2012). A review on sex differences in processing emotional signals. Neuropsychologia.

[30] Williams B, Brown T, McKenna L, Palermo C, Morgan P, Nestel D (2015). Student empathy levels across 12 medical and health professions: an interventional study. J of Compassionate Health Care.

[31] Bahadur G, Arjyal A, Douglas A, Subedi M, Gongal R. A quantitative evaluation of empathy using JSE-S Tool, before and after a medical humanities Module, amongst first-year medical students in Nepal. BMC Med Educ. 2022;22. 10.1186/s12909-022-03188-y PMC8903097 35260157

[32] Chen P-J, Huang C-D, Yeh S-J (2017). Impact of a narrative medicine programme on healthcare providers’ empathy scores over time. BMC Med Educ.

[33] Yang K-T, Yang J-H (2013). A study of the effect of a visual arts-based program on the scores of Jefferson scale for physician empathy. BMC Med Educ.

[34] Schwartz B, Horst A, Fisher J, Michels N, Van Winkle L. Fostering Empathy, Implicit Bias Mitigation, and compassionate behavior in a medical humanities Course. Volume 17. INTERNATIONAL JOURNAL OF ENVIRONMENTAL RESEARCH AND PUBLIC HEALTH; 2020. 10.3390/ijerph17072169 PMC7177225 32218103

[35] Van Winkle L, Thornock B, Schwartz B, Horst A, Fisher J, Michels N. Critical reflection on required service to the community propels prospective medical students toward higher empathy, compassion, and bias mitigation but are these gains sustainable? Volume 9. FRONTIERS IN MEDICINE; 2022. 10.3389/fmed.2022.976863 PMC9500161 36160142

[36] Yang N, Xiao H, Cao Y, Li S, Yan H, Wang Y (2018). Does narrative medicine education improve nursing students’ empathic abilities and academic achievement? A randomised controlled trial. J Int Med Res.

