# Supplementary material for: Educational efficacy of medical humanities in empathy of medical students and healthcare professionals: a systematic review and meta-analysis
Source: BMC Med Educ. 2023 Dec 6;23:925. doi: 10.1186/s12909-023-04932-8 (PMC10698992; doi:10.1186/s12909-023-04932-8)
Supplement: Supplementary file 2 — Supplementary Material 2: Appendix 2 Best Evidence Medical Education (BEME) coding scheme for strength of evidence and Kirkpatrick-based outcomes [file 12909_2023_4932_MOESM2_ESM.docx]

**Appendix 3**

| Author name (year) | Study location | Design | Type of intervention | Sample descriptions | Measurement Tool | Sample Size | Intervention time | Gender in total | Empathy score, mean(SD) |
| --- | --- | --- | --- | --- | --- | --- | --- | --- | --- |
| Bahadur et al.(2015) | Nepal | Single arm pre post study | Course AND Expose to care practice AND Reflective writing practice | First-year medical students | JSE-S | 65 | 8 weeks | Males: 32 Females:30 | Pre-: 105.52(10.45) Post-: 116.29(9.02) |
| Johanna et al.(2004) | USA | Single arm pre post study | Course | First-year medical students | ECRS | 16 | 3 Months | Males: 6 Pre-: 53.8(19.3) Post-: 62.3(17.9) Females: 10 Pre-: 59.0(21.4) Post-: 72.9(16.9) | Pre-: 92.3(8.2) Post-: 94.6(8.9) |
| Xue et al.(2023) | China | Single arm pre post study, RCT | Course AND Reflective writing practice | Nursing students | JSPE-NS | 85 Intervention group: 43 Control group: 42 | 12 Months | No reported | All-Post: I-G:99.4(15.7) C-G:92.2(14.6) I-Group: Pre-: 89.6(14.0) Post-: 99.4(15.7) C-G: Pre-: 88.7(11.9) Post-: 92.2(14.6) |
| Michal et al.(2020) | Israel | Single arm pre post study | Course | Medical students | JSPE-S | Cohort 1: 91 -panel-style of the admission process -first year of course content Cohort 2: 86 -Multiple OSCE-style of the admission process -first year of course content Cohort 3: 85 -Multiple OSCE-style of the admission process three year of course content | 3 years | All Males: 147 Pre-: 114.54(11.33) Post-: 112.13(13.99) All Females: 114 Pre-: 114.11(11.38) Post-: 113.78(14.42) Cohort 1 and Cohort 2: 177 Males: 109 Pre-: 113.87(11.99)Post-:  112.31(13.59) Females: 68 Pre-: 117.72()10.69 Post-:  111.48(14.81) Cohort 3: 85 Males: 38 Pre-: 116.29(9.30) Post-:  111.67(15.19) Females: 46 Pre-: 118.47(11.43) Post-: 117.97(12.86) | Pre-: 114.40(11.32) Post-: 112.75(14.19) |
| Chen et al.(2017) | Taiwan, China | Single arm pre post study | Course AND Reflective writing practice | Medical Staff | JSE-HP | 142 | 2 Months | Males:  Pre-(13): 108.6(5.0) Post-(15):106.7(5.3) 1.5years(12):109.0(3.1) Females:  Pre-(97): 111.4(1.5) Post-(85):117.9(1.6) 1.5years(90):116.6(1.6) | Pre-(110): 111.1(1.4) Post-(100): 116.2(1.6) 1.5years(90): 116.0(1.6) |
| Yang et al.(2013) | Taiwan, China | Single arm pre post study | Exposure to visual art | Medical students AND postgraduate residents | JSPE | 113 | 4 Months | Males: 77 Pre-: 110.29(10.17) Post-: 109.99(11.54) Females: 33 Pre-: 112.36(10.52) Post-: 114.36(11.20) | Pre-: 110 110.92(10.27) Post-:110 111.30(11.57) |
| Cédric et al.(2020) | France | RCT | Course AND Reflective writing practice | Medical students | JSPE-MS | Control: 117 Balint groups: 125 Narrative medicine: 120 | 2 Months | Males: Control(42): 107.0(11.5) Balint groups(53): 109.3(9.5)  Narrative medicine(54): 108.0(10.2) Females:  Control(75): 111.8(11.8) Balint groups(125): 112.3(8.6) Narrative medicine(66): 110.7(9.3) | Control:  110.1(11.9) Balint groups: 111.0(9.1) Narrative medicine:  110.7(9.3) |
| Chen et al.(2022) | Taiwan, China | RCT | Narrative/Storytelling | Medical students | JSPE | Control: 138 Intervention: 69 | 9 Months | No reported | Control:  69.4(11.3) Intervention:  69.7(11.9) |
| Brian et al.(2020) | USA | Single arm pre post study | Course AND Expose to care practice AND Reflective writing practice | Medical students | JSE | 34 | 4 Months | No reported | Pre-: 5.75(0.1) Post-: 6.05(0.09) |
| Haley et al.(2018) | USA | Single arm pre post study | Course AND Reflective writing practice | Medical students | Self-made questionnaire(contains 9 items) | 25 | 1 years | No reported | Pre-: 25 3.25(0.42) Post-:22 3.82(062) |
| Lon J et al.(2021) | USA | Single arm pre post study | Course | Medical students | JSE | 60 | 4 Months | No reported | Pre-:  109.10(1.28) Post-: 112.22(1.07) 从图形中获取数据 |
| Yang et al.(2018) | China | Single arm pre post study, RCT | Course AND Expose to care practice AND Reflective writing practice | nursing students | JSE | 163 Group 1(52): No education in narrative medicine Group 2(56): theoretical education Group 3(55):  theoretical education and clinical practice education | 30Months | No reported | T1(pre-): G1:104.08(12.43) G2:104.59(13.48) G3:104.42(14.11) T2(theoretical education ends) G1:104.06(11.75) G2:107.45(13.34) G3:107.07(14.08) T3(clinical practice education ends) G1:104.79(11.82) G2:107.91(13.01) G3:110(13.30) T4(After 0.5 years) G1:105.25(11.94) G2:108.04(13.21) G3110.11(13.30) T5(After 1 years) G1:105.73(11.94) G2:108.27(13.09) G3:110.62(13.22) T6(After 1.5 years) G1:103.85(12.00) G2:107.57(12.83) G3:109.98(13.37) |
| Zhao et al.(2023) | China | Single arm pre post study | Course | Medical Staff | JSPE-S | 116 | 2 Months | No reported | Pre-:  110.6(12.1) Post: 122.6(9.0) |
| Saeideh et al.(2020) | Iran | Single arm pre post study, RCT | Course AND Reflective writing practice | Medical students | JSPE | 135 Control: 66 Intervention: 69 | 3 Months | No reported | C-G: Pre-: 75.86(8.50) Post: 76.35(7.99) I-G: Pre-: 73.90(8.59) Post: 94.90(4.47) |
| Lu et al.(2023) | China | Single arm pre post study | Course | nursing | The Interpersonal Response Index Scale | 101 | 6 Months | No reported | Pre-: 21.64(2.3) Post: 26.71(3.1) |
