# Supplementary material for: Educational efficacy of medical humanities in empathy of medical students and healthcare professionals: a systematic review and meta-analysis
Source: BMC Med Educ. 2023 Dec 6;23:925. doi: 10.1186/s12909-023-04932-8 (PMC10698992; doi:10.1186/s12909-023-04932-8)
Supplement: Supplementary file 3 — Supplementary Material 3: Appendix 3 Characteristics of included studies [file 12909_2023_4932_MOESM3_ESM.doc]

**Appendix 4**

| **Author name (year)** | **BEME** | **Kirkpatrick-based outcome levels** |
| --- | --- | --- |
| Bahadur et al.(2015) | 3 | 2a |
| Johanna et al.(2004) | 3 | 2a |
| Xue et al.(2023) | 4 | 2b |
| Michal et al.(2020) | 4 | 2a |
| Chen et al.(2017) | 4 | 2b |
| Yang et al.(2013) | 3 | 2b |
| Cédric et al.(2020) | 4 | 2a |
| Chen et al.(2022) | 3 | 2a |
| Brian et al.(2020) | 3 | 2a |
| Haley et al.(2018) | 3 | 2a |
| Lon J et al.(2021) | 3 | 2a |
| Yang et al.(2018) | 4 | 2b |
| Zhao et al.(2023) | 3 | 2a |
| Saeideh et al.(2020) | 4 | 2a |
| Lu et al.(2023) | 3 | 2a |
