# Supplementary material for: Educational efficacy of medical humanities in empathy of medical students and healthcare professionals: a systematic review and meta-analysis
Source: BMC Med Educ. 2023 Dec 6;23:925. doi: 10.1186/s12909-023-04932-8 (PMC10698992; doi:10.1186/s12909-023-04932-8)
Supplement: Supplementary file 4 — Supplementary Material 4: Appendix 4 BEME and Kirkpatrick-based results [file 12909_2023_4932_MOESM4_ESM.doc]

**Appendix 1**

WOS：TS = ("medical humanities" OR "health humanities" OR "Narrative medicine") AND (Empathy OR compassion OR compassionate)

Pubmed : ("medical humanities"[Title/Abstract] OR "health humanities"[Title/Abstract] OR "Narrative medicine"[Title/Abstract]) AND (Empathy[Title/Abstract] OR compassion[Title/Abstract] OR compassionate[Title/Abstract])

(Narrative medicine[MeSH Terms]) AND (Empathy[Title/Abstract] OR Compassion[Title/Abstract] OR Compassionate[Title/Abstract])

(Empathy[MeSH Terms]) AND ("medical humanities"[Title/Abstract] OR "health humanities"[Title/Abstract] OR "Narrative medicine"[Title/Abstract])

Embase : ('medical humanities':ti,ab,kw OR 'health humanities':ti,ab,kw OR 'narrative medicine':ti,ab,kw) AND (empathy:ti,ab,kw OR compassion:ti,ab,kw OR compassionate:ti,ab,kw)

EBSCO-ERIC:

ALL TEXT="medical humanities" and ALL TEXT=Empathy

ALL TEXT="medical humanities" and ALL TEXT=compassion

ALL TEXT="medical humanities" and ALL TEXT=compassionate

ALL TEXT="health humanities"and ALL TEXT=Empathy

ALL TEXT="health humanities"and ALL TEXT=compassion

ALL TEXT="health humanities"and ALL TEXT=compassionate

ALL TEXT="Narrative medicine" and ALL TEXT=Empathy

ALL TEXT="Narrative medicine" and ALL TEXT=compassion

ALL TEXT="Narrative medicine" and ALL TEXT=compassionate
